# Fiji-Based Tool for Rapid and Unbiased Analysis of SA-β-Gal Activity in Cultured Cells

**DOI:** 10.3390/biom13020362

**Published:** 2023-02-14

**Authors:** Adam Krzystyniak, Agata Gluchowska, Grazyna Mosieniak, Ewa Sikora

**Affiliations:** Laboratory of Molecular Bases of Aging, Nencki Institute of Experimental Biology, Polish Academy of Sciences, 02-093 Warsaw, Poland

**Keywords:** senescence, Fiji macro, SA-β-galactosidase

## Abstract

Normal cells under stressful conditions such as DNA damage or excessive mitogenic signaling may undergo senescence, which is associated with cell cycle arrest and induction of a proinflammatory phenotype. Accumulation of senescent cells may contribute to the shortening of the life span by accelerating aging and promoting chronic diseases. Cytochemical detection of the senescence-associated β-galactosidase (SA-β-gal) activity with 5-bromo-4-chloro-3-indolyl β-D-galactopyranoside (X-gal) is a widely recognised marker of cell senescence. However, its simplicity and cost effectiveness lead to limitations in quantification, which is usually limited to manual counting of the positive cells. In order to address those limitations, we developed a Fiji-based macro extension that performs automatic and unbiased analysis of the integrated density of SA-β-gal specific signal. Our tool is not only faster than manual counting but also provides extra resolution compared to the manual methods. Our macro extension could be a valuable tool in any senescence research laboratory.

## 1. Introduction

Cellular senescence is a phenomenon in which cells undergo profound phenotypic changes upon different types of stimuli such as DNA damage, oxidative stress (stress-induced premature senescence, SIPS), exhaustion of replication potential (replicative senescence) or expression of oncogenes (oncogene-induced senescence; OIS) [1]. Apart from irreversible cell cycle arrest and proinflammatory senescence-associated secretory phenotype (SASP), one of the most widely recognised markers of senescence is increased activity of β-galactosidase detectable at pH 6.0, termed “senescence-associated β-galactosidase” (SA-β-gal) [2]. Senescent cells accumulate with age and are believed to be one of the critical contributors to age-associated diseases such as cardiovascular disease, cancer, arthritis, osteoporosis, type 2 diabetes and Alzheimer’s disease. They contribute to those conditions by inducing systemic inflammation and limiting regenerative capacities [3]. Since cell senescence is considered to be the culprit of aging, SA-β-gal has been extensively used for demonstrating age-associated changes at the cellular level that promote diseases and for testing various anti-aging compounds and interventions [4]. It is important to note, however, that β-gal activity at pH 6.0 is not exclusive to the senescent phenotype. Thus, when used for identification of senescent cells, it should be used in tandem with other markers of senescence such as expression of specific proteins (p16, p21) and indicators of cell cycle arrest (lack of BrdU or Ki67) [5]. Currently, there are two methods of SA-β-gal activity detection that are common in cell senescence research, namely fluorescence based and cytochemical. Both of the procedures involve enzymatic cleavage of the β-galactosidase substrate in pH = 6.0. An example of a fluorescence-based method substrate is C12FDG [6]; however, there are other commercially available substrates such as SPiDER-ßGall [7]. For cytochemical SA-β-gal detection 5-bromo-4-chloro-3-indolyl β-D-galactopyranoside (X-gal) [4] is the most common; however, 2-Nitrophenyl β-D-galactopyranoside (ONPG) is also used for quantification of the enzyme activity in lysates [8]. Although fluorescence-based methods enable SA-β-gal activity analysis in fixed and living cells in a qualitative and quantitative manner through flow cytometry or fluorescent microscopy, it is relatively expensive and requires specialized equipment. Cytochemical methods are limited to fixed cells; however, their simplicity and cost effectiveness often makes them attractive alternatives. One of the biggest limitations of cytochemical detection of SA-β-gal in cells is quantification, which is usually performed by manually counting the positive cells. Not only is manual counting very time-consuming, but it also may vary between investigators due to subjective classification of positive cells. Furthermore, counting positive cells is, in principle, less sensitive than analysing signal intensity due to lower resolution. Analysing only the percentage of positive SA-β-gal cells deprives the assessment of additional dimensions. To address the limitations of the cytochemical method associated with quantification of SA-β-gal signal, we are proposing a tool which we developed and found particularly useful in our research. The tool is a Fiji (platform designed to facilitate image analysis for the scientific community [9]) macro extension designed to measure SA-β-gal in microscopic images from cell cultures by calculating the integrated density of cleaved X-Gal specific signal. This method is not designed to identify senescent cells; rather, it allows for fast, automatic and unbiased quantification of SA-β-gal activity, which might significantly accelerate realization of tasks associated with cell senescence research. We believe that the tool designed by us may complement other available tools allowing the digitally enhanced quantification of senescence.

## 2. Materials and Methods

### 2.1. Cells Culture

Human vascular smooth muscle cells (VSMCs) were purchased from ATCC (normal diploid cells derived from young males, at least from three different donors) and were cultured in a vascular cell basal medium (ATCC) supplemented as defined by the manufacturer and kept in a humidified atmosphere (37 °C and 5% CO_2_ in the air). Cells were seeded at a density of 3000/cm^2^ and passaged every 3–4 days. To induce senescence, 24 h after seeding, cells were treated with 1 µM doxorubicin (for 2 h) or with 150 µM H_2_O_2_ (not washed after treatment) and cultured for 3 or 7 days. 

### 2.2. SA-β-Gal and DAPI Staining

Detection of SA-β-Gal was performed according to the method described by Dimri et al. [1]. Briefly, cells were fixed with 2% formaldehyde, 0.2% glutaraldehyde in PBS, washed, and exposed overnight at 37 °C to a solution containing 1 mg/mL 5-bromo-4-chloro-3-indolyl-b-D-galactopyranoside, 5 mM potassium ferrocyanide, 5 mM potassium ferricyanide, 150 mM NaCl, 2 mM MgCl_2_, and 0.02 M phosphate buffer, pH 6.0. All of the used agents were purchased from Sigma-Aldrich. Imaging cells were embedded in 4ul of mounting medium with DAPI (Abcam) and mounted on glass slides. Images were taken at least 10 min after embedding the cells in the medium with transmitted light or fluorescence (excitation 340–380 nm, emission 435–485 nm) using the Nikon Eclipse Ti-U fluorescent microscope and Nikon Digital Sight DS-U3 camera (Nikon) with 20× objective at resolution 2560 × 1920 pixels and exposure set at 15 ms (BF images) with 30% of maximum lamp intensity. All results were compared to control (untreated cells), which were fixed and stained for SA-β-Gal and with DAPI 48 h after seeding. To ensure imaging consistency, all cells used for analysis were fixed and stained simultaneously using the same stock solutions and working solutions. To decrease the possible effect of variability in equipment performance on the SA-β-gal positive signal intensity, care was taken to photograph all slides (negative control (untreated cells cultured for 48 h) and treatment variants (doxorubicin or H_2_O_2_)) from one donor within one imaging session. SA-β-gal staining analysis is sensitive to imaging and staining artifacts such as air bubbles, precipitated crystals or dark objects such as non-transparent debris. During image analysis, care was taken not to obtain images of visual fields that would include such objects.

### 2.3. SA-β-Gal Staining Analysis

For counting trials, we analysed at least 100 cells from three independent biological repeats using manual and automatic methods.

1. Automatic method

**Figure 1:**
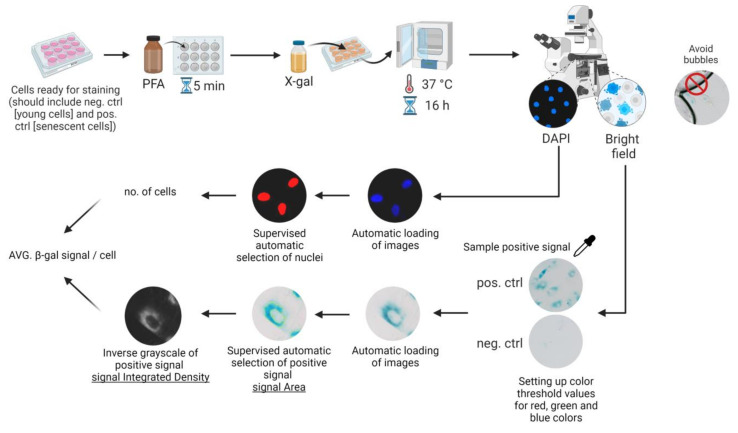
Schematic representation of SA-β-gal positive signal analysis in cells using macro extension. Staining of cells with X-gal and DAPI allows collection of overlapping brightfield (SA-β-gal signal) and fluorescence (DAPI) images of cells. Images are analysed with a macro consisting of two separate modules. The first module performs automatic SA-β-gal positive signal analysis in multiple pictures based on pre-set parameters (parameters set during colour thresholding). The second module automatically estimates cell numbers based on DAPI nuclear staining. Resulting data may be used for calculation of area of the SA-β-gal positive signal or integrated density per cell.

In the automatic method, the images were analysed with macro extension (Supplementary Materials, beta-gal analyzer.ijm) according to the manual which can be found in the Supplementary Material together with the macro code (Supplementary Materials). For the development of this tool, we used the Fiji platform providing image analysis solutions for the scientific community [9]. The macro extension has been designed to automatically process multiple images of cells from monolayer cell culture stained for SA-β-gal activity with X-gal and nuclei with DAPI. The extension performs a series of image-analysis steps, which result in the creation of selection around areas of the image consisting of pixels displaying an amount of blue, green, and red above the experimentally determined colour threshold (Figure 1). The baseline colour signal is set on the control cells (cells on a low passage number cultured in low confluency for up to 48 h), which are supposed to have background activity of SA-β-gal. For resolution between the treatment and control group, there should be a pronounced difference in the intensity of SA-β-gal staining between cells with background SA-β-gal activity and senescent cells. Based on our experience with doxorubicin and H2O2-treated VSMCs as a model of senescence, we arbitrarily assumed that signal from control cells should be between 0 and 10% of that obtained from senescent cells. Working with different cells and/or different types of senescence induction or staining conditions may require revision of that value. For more details on the setting up a colour threshold for SA-β-gal signal analysis, please refer to the manual—in particular, the troubleshooting section (Supplementary Materials, Section 4, beta-gal analyzer). Integrated density (ID) is our parameter of choice for describing SA-β-gal signal. However, Fiji provides multiple parameters which could be alternatively used, including area of the signal, mean grey value and raw integrated density. ID as a sum of values of all pixels (in unscaled images, this is considered the raw integrated density) in the selected area and it provides information regarding the integration of potential changes in the area of the signal together with its intensity. We do not use deconvolution to analyse signal intensity in our tool. To simplify the measurement, the extension is designed to analyse the intensity of the pixel that has been recognised as a SA-β-gal positive signal (based on pre-set threshold values) in inverted grayscale. ID values could be translated into an increase in the size of the SA-β-gal filled cytoplasmic compartment and expression of SA-β-gal enzyme, respectively. The extension automatically detects and processes all images in the given catalogue. However, it is carried out under supervision. After analysis of each individual image, the user is asked for confirmation that the image was properly analysed, decreasing the likelihood of macro mistake or analysing images containing objects such as air bubbles that may influence the analysis results, mentioned in the “SA-SA-β-gal and DAPI staining” subsection. The macro extension is equipped with a module that allows semi-automatic counting of DAPI-stained nuclei. It creates selection around DAPI positive elements under supervision of the user and calculates the number of nuclei in the ROI and area of each nuclei. Estimation of cell numbers in the ROI allows for calculation of the ID per cell, which we use as a final value for further analysis.

2. Manual method

A manual count was performed by a trained investigator blinded to the experimental conditions. Manual count was considered the standard method, which was used for comparison with the automatic procedure.

### 2.4. Statistical Analysis

Statistical analysis was performed using a two-tailed Student’s *t* test to examine the differences between the two groups. Data are presented as a mean ± SD. A value of *p* ≤ 0.05 was considered statistically significant (* *p* ≤ 0.05, ** *p* ≤ 0.01, *** *p* ≤ 0.001). All graphs show the mean results from at least three independent experiments.

## 3. Results

**Figure 2:**
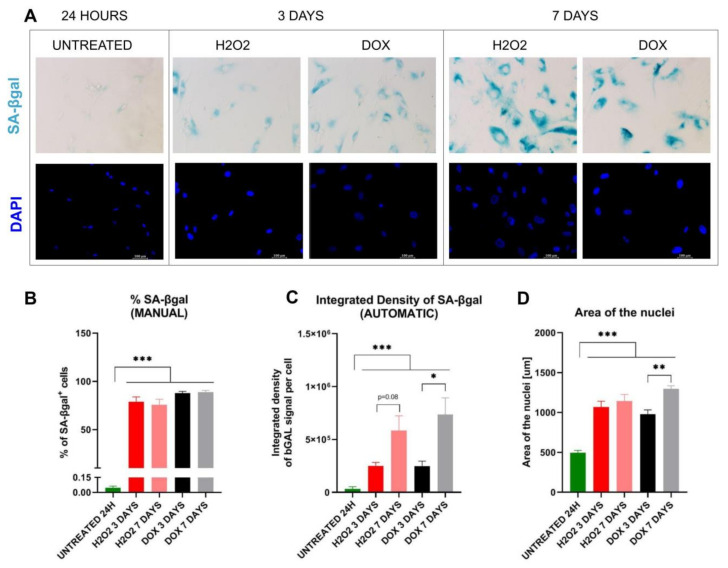
Representative data showing capabilities of the macro extension to analyse SA-β-gal signal in human VSMC cells. VSMC cells were cultured in presence of doxorubicin (1 µM for 2 h) or H_2_O_2_ (single dose of 150 µM). On days 3 and 7, cells were fixed and stained with X-gal for SA-β-gal activity and with DAPI for nuclear staining. Panel A depicts representative images from the stained cells (**A**). (**B**) Results of manual and (**C**) automatic SA-β-gal activity analysis are presented alongside estimated differences in the area of the nuclei of the cells which increase in senescent cells (**D**). Results are mean with SEM from N = three biological replicates. Statistical analysis performed using Student’s *t*-test, * *p* ≤ 0.05, ** *p* ≤ 0.01, *** *p* ≤ 0.001.

For the purpose of this work, we performed a set of experiments aimed at evaluating our macro extension. For the evaluation round, we have used human vascular smooth muscle cells (VSMCs). VSMCs significantly upregulate SA-β-Gal activity upon senescence induction. Moreover, due to the fact that senescent VSMCs are associated with artherosclerotic plaque formation, there are well-established protocols for senescence induction in that cell type [8,9]. The model is based on senescence induction through treatment of the cells with doxorubicin or H_2_O_2_ (Figure 2A). For evaluation purposes, the cells, after incubation with X-gal at pH = 6.0 for 16 h in 37 °C, were analysed manually (Figure 2B) and automatically using macro extension (Figure 2C).

### 3.1. Macro Extension Enables Identification of Samples with SA-β-gal Positive Cells from Control Cells

We found that the macro extension accurately distinguishes control samples from samples with elevated SA-β-gal expression regardless of senescence inducer (doxorubicin or H_2_O_2_) (untreated 24 h vs. H_2_O_2_-treated 3d, *p* ≤ 0.001; untreated 24 h vs. H_2_O_2_-treated 7d, *p* ≤ 0.001; untreated 24 h vs. DOX-treated 3d, *p* ≤ 0.001; untreated 24 h vs. DOX-treated 7d, *p* ≤ 0.001) (Figure 2B). The results are comparable to those obtained using manual counting of SA-β-gal positive cells (untreated 24 h vs. H_2_O_2_-treated 3d, *p* ≤ 0.001; untreated 24 h vs. H_2_O_2_-treated 7d, *p* ≤ 0.001; untreated 24 h vs. DOX-treated 3d, *p* ≤ 0.001; untreated 24 h vs. DOX-treated 7d, *p* ≤ 0.001) (Figure 2C).

### 3.2. Macro Extension Enables Detection of Differences in SA-β-gal Signal Intensity between Samples with SA-β-gal Positive Cells

Here, we compare standard SA-β-gal positive vs negative discrimination with our approach. In these experimental settings, the manual method failed to detect more subtle differences between early and late senescent cells (3 days and 7 days post-treatment with doxorubicin and H_2_O_2_) (Figure 2C). Our macro extension was able to reveal significant differences between 3 and 7 days in doxorubicin post-treatment cells (H_2_O_2_-treated 3d vs. H_2_O_2_-treated 7d, *p* = 0.08; DOX-treated 3d vs. DOX-treated 7d, *p* = 0.042) (Figure 2B) because it can distinguish cells with low activity of SA-β-gal from those with high SA-β-gal activity. We confirmed that higher resolution of the SA-β-gal signal reflects differences in other features of cell senesce phenotype, such as increasing area of the nucleus [10,11]. Here, we found that all senescent cells have a larger area of the nuclei compared to control cells and that this parameter significantly increases 7 days after doxorubicin treatment compared to the 3 days’ time point (H_2_O_2_-treated 3d vs. H_2_O_2_-treated 7d, *p* = 0.52; DOX treated 3d vs. DOX treated 7d, *p* = 0.008) (Figure 2D). The manual counting which we implemented did not differentiate cells to SA-β-gal positive with low or high signal intensity.

### 3.3. Macro Extension Significantly Facilitates Analysis of Multiple Images of Cells Stained for SA-β-gal Activity

Most importantly, however, there was a considerable difference in the time necessary for analysis of cells using both methods. On average, manual analysis of a set of five images required (depending from the number of cells on the image) approx. 10 min (including manual nuclei counting), whereas with our macro extension, it was possible to carry it out within approx. 30 s in the hands of a trained investigator. We would like to underline that we did not include the time necessary for the analysis of the data set using other tools designed for digitally enhanced analysis in the comparison [12,13].

## 4. Discussion

SA-β-gal activity, although not fully specific, is the most commonly used marker of senescence [5]. Here, we present a tool developed on the Fiji platform that allows rapid screening of changes in SA-β-gal activity without investigators’ bias associated with subjective classification of positive cells. Furthermore, the ability of our tool to detect differences in the intensity of the staining provides additional sensitivity in detecting more subtle differences between various types of cell senescence inducers. We found that our macro extension is able to detect differences in the level of SA-β-gal activity better than standard SA-β-gal positive vs negative discrimination-based manual counting. Interestingly, the increase in SA-β-gal activity detected by our tool between senescence-induced VSMCs cultured for 3 days and 7 days reflected the previously described, time-dependent development of the senescence phenotype. It has been shown by us and others that within the time window used by us in this work, cell senescence deepens [14,15]. Higher resolution obtained with our tool might translate into a better understanding of molecular mechanisms associated with the cell senescence phenomenon and more accurate assessment of substances with senolytic or senomorphic properties.

### 4.1. Comparisons with Other Methods

There are at least two other tools providing digitally enhanced manual analysis of SA-β-gal positive cells. One developed on the ImageJ platform uses colour thresholding to differentiate positive from negative SA-β-gal cells [12], whereas the other one developed in MATLAB combines manual cell marking with bGAL signal analysis [13]. In contrast to the existing tools, our macro performs simplified automatic detection of SA-β-gal signal on multiple images with limited users’ input, allowing for quantification of signal intensity per cell. We are not aware of any other tool (including commercial tools) that would perform a similar type of analysis to that which we describe here in terms of combining speed of analysis and principle associated with integrated density measurement. We believe that using our tool and method for quantification of bGAL activity could not only facilitate it but also provide an additional layer of resolution to existing algorithms and tools used to identify substances that affect cell senescence.

### 4.2. Limitations of the Method

Despite the fact that we believe the tool that we developed could be very useful in everyday laboratory practice, we recognized several critical issues which should be taken into account when using the tool. SA-β-gal staining is influenced to a certain degree by staining conditions such as time of sample incubation with the X-gal, the preparation of the substrate, etc. For this reason, the time of staining should be experimentally determined in order to avoid saturation of the SA-SA-β-gal signal, particularly in senescent cells or positive control. Once saturated, it decreases the resolution of the method. Finally, it is important to image the stained cells with the same setup, both hardware (optics and camera) and software. Sensitivity of the SA-SA-β-gal staining to staining conditions limits this tool to comparison between different treatment groups of the SA-SA-β-gal staining preferentially performed simultaneously on all of the samples. This is probably true for any type of β-gal staining. Our method is suitable for dense cultures since it calculates the average β-Gal signal per cell, eliminating the need of knowing the perimeter of each individual cell. However, due to the fact that the number of cells is estimated based on the number of nuclei in the region of interest, we recommend manual counting of the number of cells in a dense multinucleated-cells culture. When the culture of multinucleated cells allows the distinguishing of nuclei from individual cells without additional staining, our tool has the ability to analyse cells with multiple nuclei (Supplementary Materials, Sections 3.a.X and 3.b.III).

## 5. Conclusions

Cellular senescence is one of the hallmarks of aging and it is becoming evident that it is a contributing factor in many age-related and chronic diseases [3]. Moreover, we and others have shown that senotherapy may alleviate age associated pathology [4,16]. Tools that would facilitate research in the field of senescence are therefore needed. Here, we provide a Fiji macro extension that we believe is a useful tool for high throughput, automatic and unbiased SA-β-gal that could complement the toolkit of any cell senescence research laboratory.

## Data Availability

Data available upon request.

## Supplementary Materials

The following supporting information can be downloaded at: https://www.mdpi.com/article/10.3390/biom13020362/s1.
