# Supplementary material for: Fiji-Based Tool for Rapid and Unbiased Analysis of SA-β-Gal Activity in Cultured Cells
Source: Biomolecules. 2023 Feb 14;13(2):362. doi: 10.3390/biom13020362 (PMC9953415; doi:10.3390/biom13020362)
Supplement: Supplementary file 1 [file biomolecules-13-00362-s001.zip › beta-gal analyzer - Fiji-based macro extension manual.pdf]

beta-gal analyzer - Fiji-based macro extension manual

by Adam Krzystyniak

Table of contents

1. Introduction
2. Installation
3. β gal analysis
    a. Setting up the color threshold
    b. Actual analysis after threshold has been set
4. Troubleshooting
5. β-gal-analyser macro code

1. Introduction

Dear user here I provide you with macro extension which was developed in order to analyze senescence associated β galactosidase signal in cell culture after staining with 5-bromo-4-chloro-3-indolyl-b-D-galactopyranoside according to materials and method section of the main manuscript. I strongly recommend anyone who would like to use that macro to have at least a basic understanding of how Fiji app works.

2. Installation

In order to install a macro extension copy the beta_gal_analyser.ijm to your hard drive. Next open fiji app (fiji may be downloaded from https://imagej.net/software/fiji/) and select Plugins-> Macros -> Install… . In the browser window select the extension which you have copied to your hard drive and click Open. This will install the macro. Note that you have to install a macro each time you close and open Fiji.

The macro requires installation of the Read and Write Excel plugin. The details about the plugin and instructions on how to instal it can be found here: https://imagej.net/plugins/read-and-write-excel

3. β gal analysis

a. Setting up the threshold

The macro uses color threshold in order to select a positive β-gal staining. The macro comes with experimentally adjusted color threshold parameters for VSMC cells and staining conditions outlined in the manuscript, however if you need to readjust the threshold for other cell type or staining conditions follow the instructions below. We assumed that signal from control cells should be between 0-10% of that obtained from senescent cells for optimal resolution between treatment and control group.

There are two ways to do it, fully manual or facilitated by in build module.

Fully manual method:

I. Open Image-> Adjust-> Color Threshold… and select sample (Fig. 1 (1))

II. Open image with high β-gal stained cells (positive control) and cells at the early passage (negative control) in Fiji. (Fig. 1 (2))

III. Select the area of the image with positive β-gal staining (Fig. 1 (3))

IV. In Color Threshold click sample (Fig. 1 (4))

V. Check if color threshold correctly selects appropriate signal, if not repeat from step I - IV or adjust Hue, Saturation and Brightness (Fig. 1 (5-6))

VI. Check if the selected values properly select the negative control. If the selection is satisfactory note the Hue, Saturation and Brightness max and min values (Fig. 1 (7)).

NOTE!! There should be a significant difference in the amount of selected positive β-gal signal between senescent cells and negative control. In our experience with VSMCs induced to senescence with doxorubicin we usually got between 0-10% of β-gal positive signal in negative control images compared to that in senescent cells (as measured by the area or Integrated Density of the signal). If you are not able to achieve significant difference in β-gal positive signal between senescent cells and negative control check troubleshooting at the end of that manual.

VII. Open Plugins-> Macros -> Edit… and select the macro

VIII. In the proper part of the macro code put the parameters noted in point VI from the Color Threshold window (Fig. 1 (8)).

IX. Save the macro (press Ctrl + S) and install it again (as in paragraph 2. Installation)

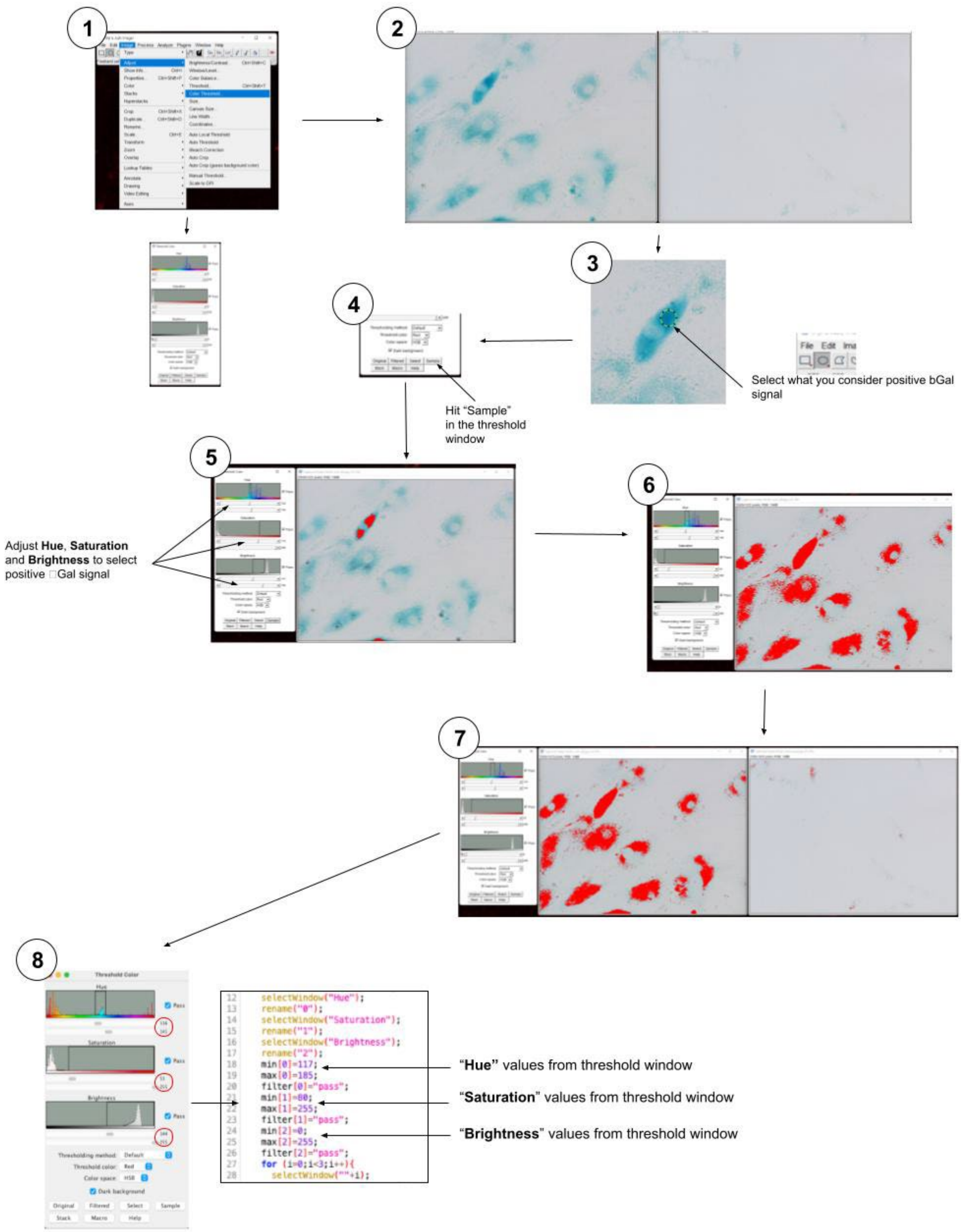

Figure 1. Schematic representation of the Threshold Setting procedure in fully manual method. No 1-8 depict consecutive steps described in point 3.a "Fully manual method"

Method facilitated by in build module:

I. After installation of the macro press "q" key and follow the instructions on the screen.
II. Once you obtain Hue, Saturation and Brightness values, Open Plugins-> Macros -> Edit… and select the macro
III. In the "Color Thresholder 2.3.0/1.53f auto-generated macro" put the parameters from Color Threshold window in the macro code
IV. Save the macro and install it again (as in paragraph 2. Installation)

X. Prepare two folders for threshold testing on your hard drive.

i. One with a set of several (5-10) images containing high β-gal stained cells (positive control) second with a set of several (5-10) images containing low β-gal stained cells (untreated control).
ii. Each of the folders should contain 2 additional folders. One containing images of β-gal staining and the other one containing DAPI staining of cells. Needless to say that images in both folders should represent staining of the same ROI.

XI. Analysis of β-gal staining

i. Press "a" key - that will initiate the macros' β-gal analysis module.
ii. Browser window will pop up.
iii. Navigate the folder where your folders for threshold testing are located
iv. Select the positive control folder with images of β-gal and hit open
v. Macro will automatically analyze every image in the folder. After every image it will ask you to confirm that everything looks OK. This visual inspection ensures that the macro does not make some unexpected mistakes. If it does reload the macro.
vi. Press "a" key again and perform steps B-E on negative control

vii. When macro is finished it will automatically generate .xlsx file with results in the folders where selected images were. Example results of βGal analysis in an image below.

	A	B	C	D	E	F	G	H	I
1	Captured Adam bGAL cont old.jpg								
2	Count	Area	Mean	StdDev	Perim.	IntDen	Median	RawIntDen	
3	1	255810	101,6511	15,16576	52519,93	26003378	98	26003378	
4									

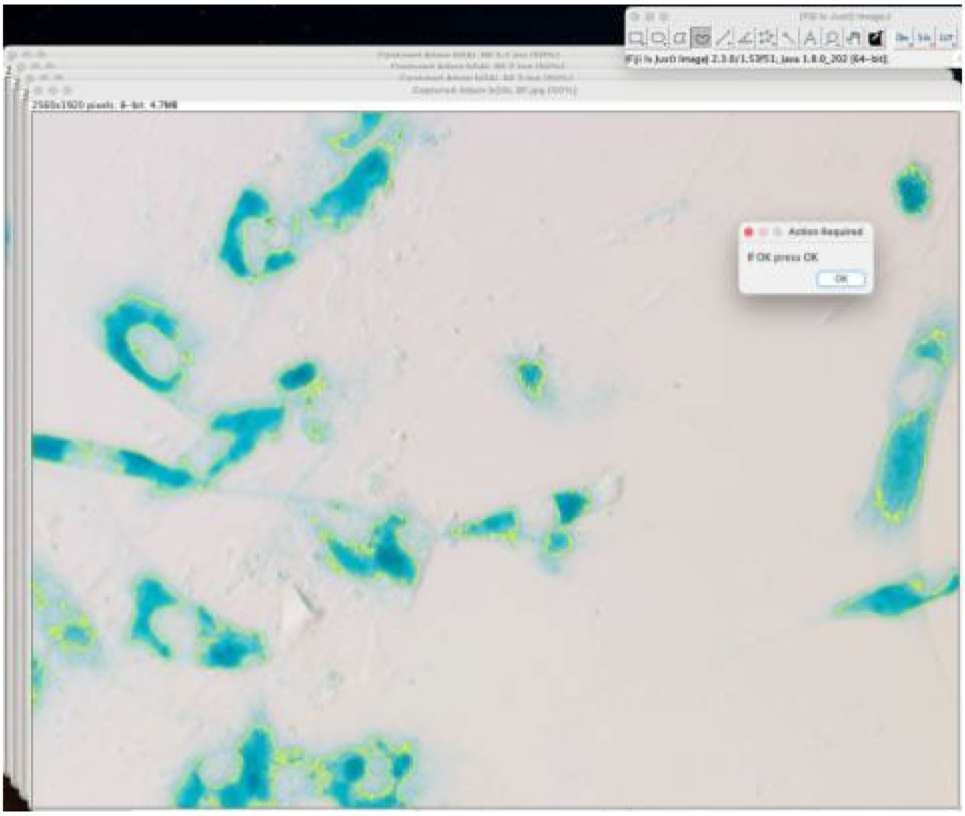

XII. Analysis of DAPI staining to estimate cell numbers

i. Press "z" key - that will initiate the macro DAPI staining cell number estimation module.
ii. Browser window will pop up.
iii. Navigate the folder where your folders for threshold testing are located
iv. Select the positive control folder with images of DAPI and hit open

v. Macro will automatically analyze every image in the folder. After every image it will ask you to confirm that automatic thresholding is done OK.

1. If you find that there are some nuclei not selected pres ctrl+shift+t in order to adjust threshold
2. If one selection encompasses more than 1 nuclei use pencil to separate nuclei (make sure to pick color of the background)

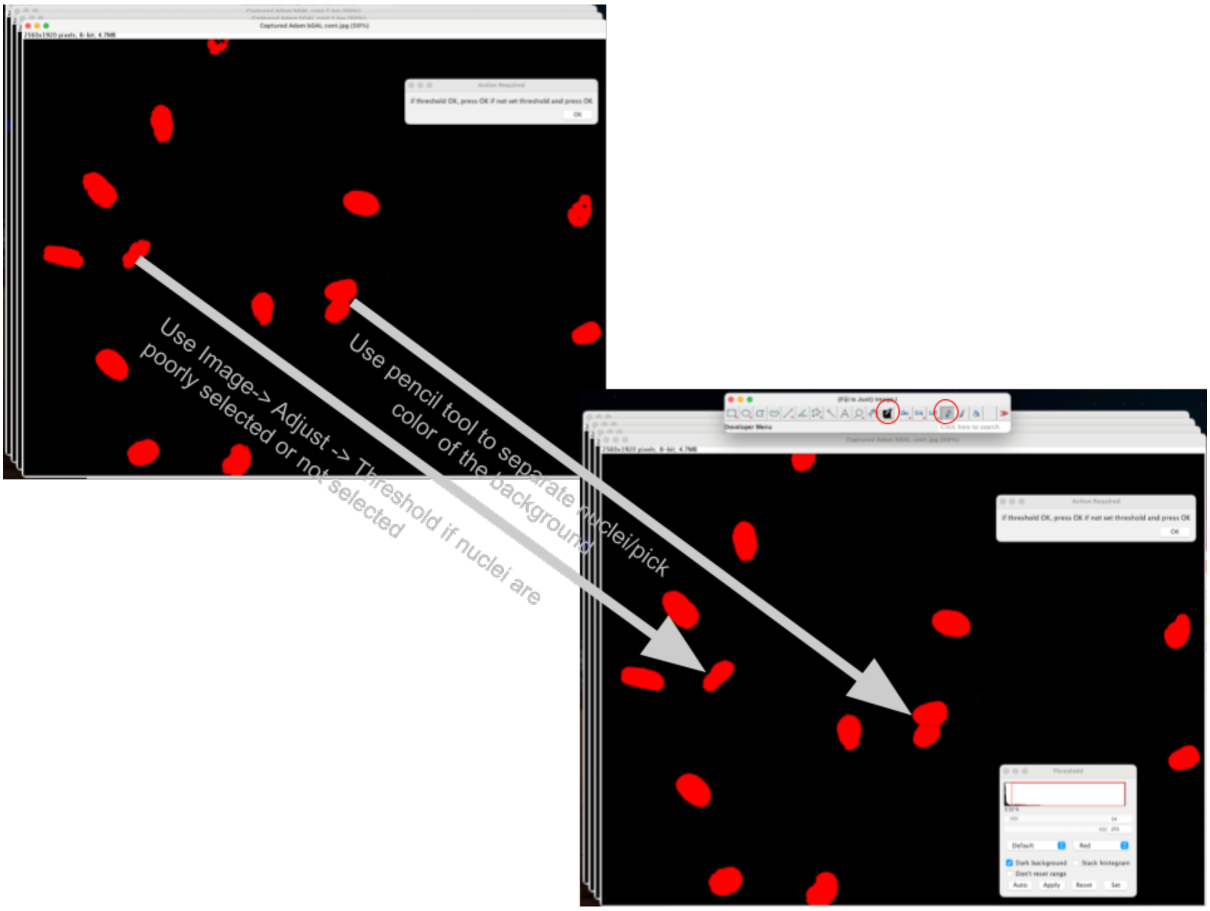

3. If the cell consist of multiple nuclei use paintbrush tool to join the nuclei together (make sure to pick the color of DAPI positive pixels)

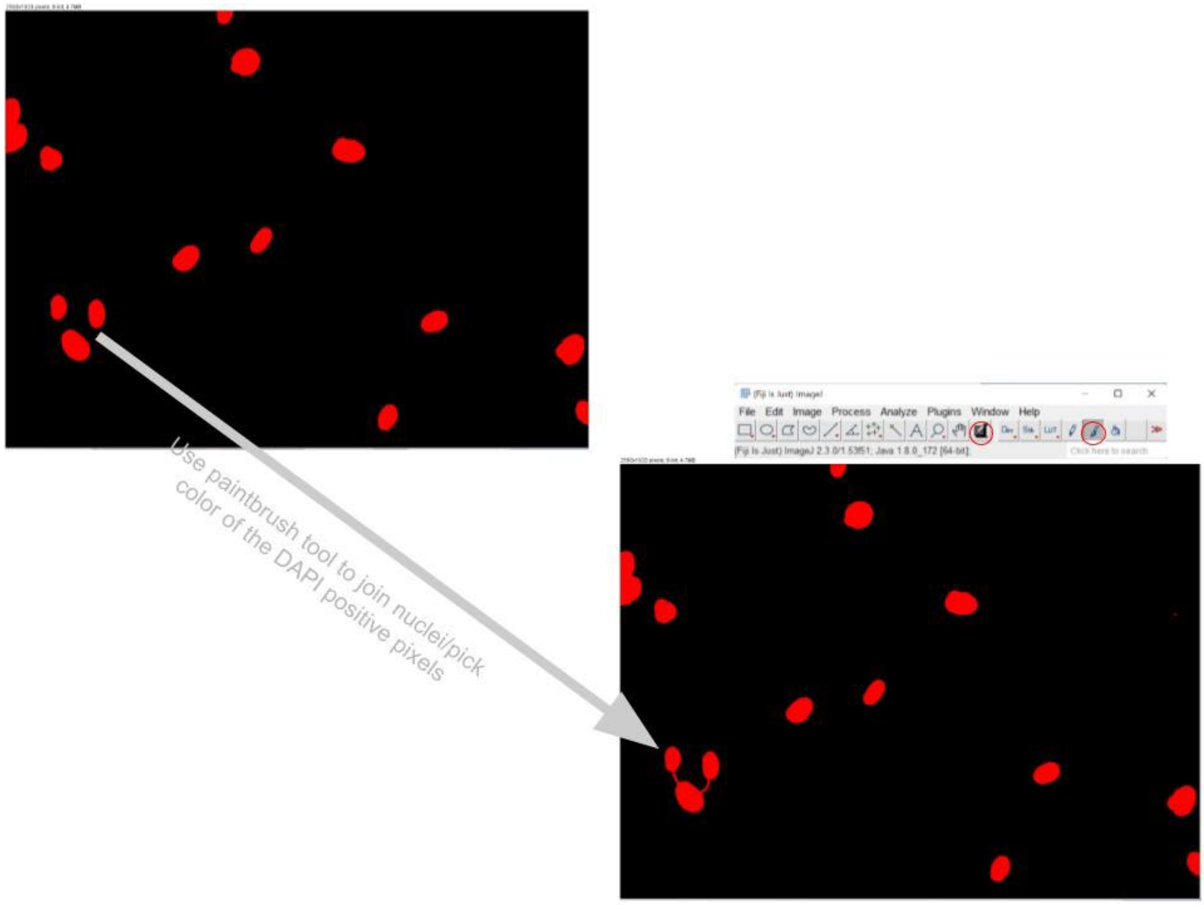

Press OK if all nuclei have been well selected

vi. press "z" key again and perform steps B-E on negative control

vii. When macro is finished it will automatically generate a .xlsx file with results in the folders where selected images were.

XIII. Calculate the Integrated Density of β-gal signal per cell. In our experience with VSMCs induced to senescence with doxorubicin we usually got between 0-10% of β-gal positive signal in negative control images compared to that in senescent cells (as measured by the area or Integrated Density of the signal). However that ratio may be different depending from cell type and conditions used.

b. Actual analysis after threshold has been set

I. For every analysis group prepare folders for the analysis on your hard drive. Each of the folders should contain 2 additional folders. One containing images of β-gal staining and the other one containing DAPI staining of cells. Needless to say that images in both folders should represent staining of the same ROI.

II. Analysis of β-gal staining

i. Press "a" key - that will initiate the macros' β-gal analysis module.
ii. Browser window will pop up.
iii. Navigate the folder where your folders for testing are located
iv. Select the folder with images of β-gal and hit open
v. Macro will automatically analyze every image in the folder. After every image it will ask you to confirm that everything looks OK. This visual inspection ensures that the macro does not make some unexpected mistakes. If it does reload the macro.
vi. Perform steps A-E on all folders containing images of β-gal staining
vii. After each folder is finished macro will automatically generate .xlsx file with results in the folders where selected images were.

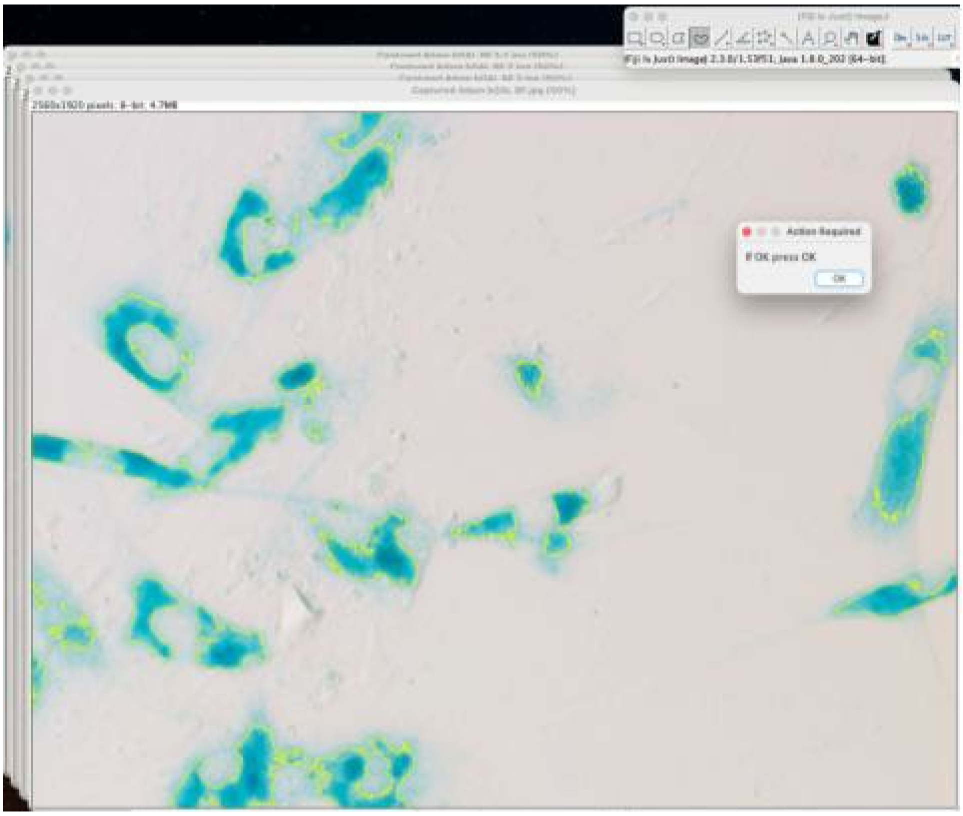

III. Analysis of DAPI staining to estimate cell numbers

i. Press "z" key - that will initiate the macro DAPI staining cell number estimation module.
ii. Browser window will pop up.
iii. Navigate the folder where your folders for testing are located
iv. Select the folder with images of DAPI and hit open
v. Macro will automatically analyze every image in the folder. After every image it will ask you to confirm that automatic thresholding is done OK.
    1. If you find that there are some nuclei not selected pres ctrl+shift+t in order to adjust threshold
    2. If one selection encompasses more than 1 nuclei use pencil to separate nuclei (make sure to pick color of the background)

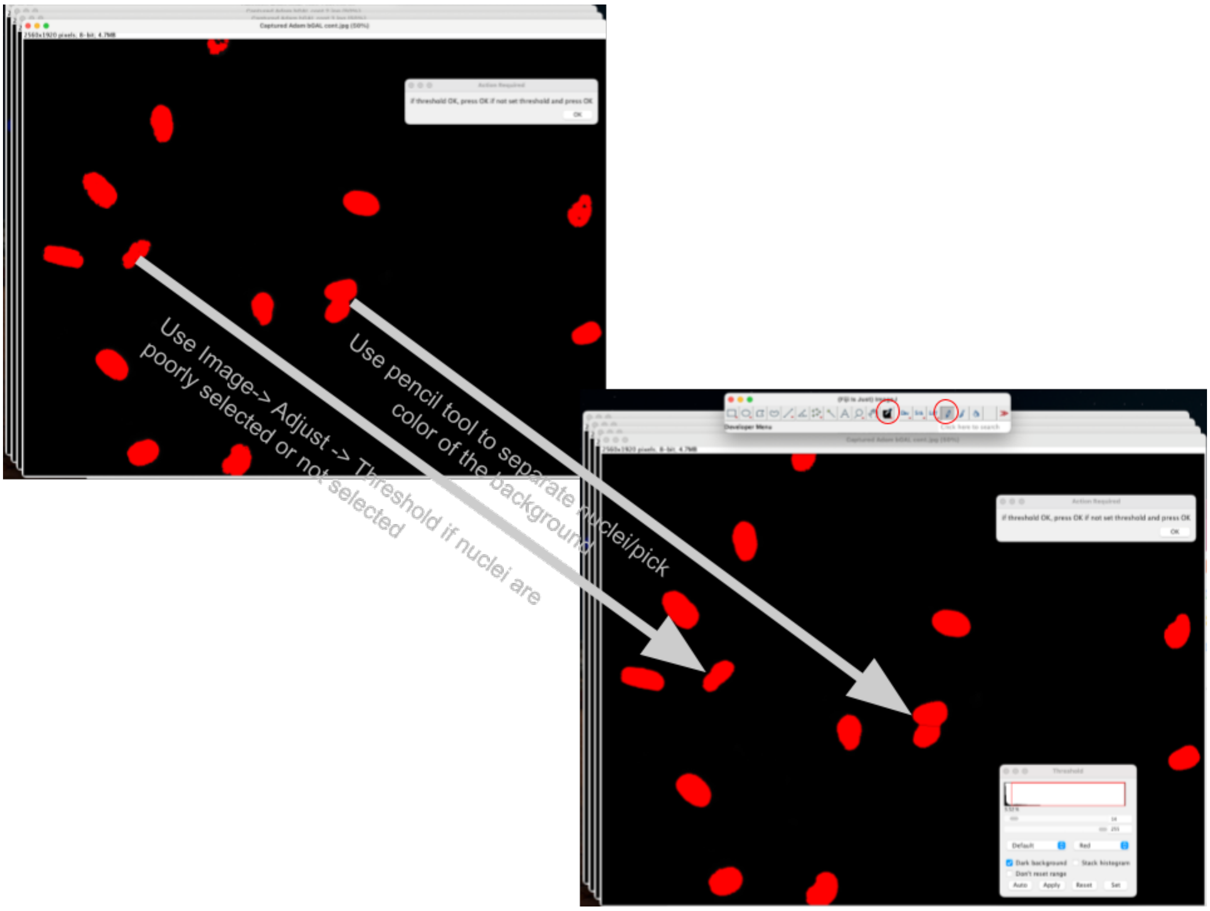

3. If the cell consist of multiple nuclei use paintbrush tool to join the nuclei together (make sure to pick the color of DAPI positive pixels)

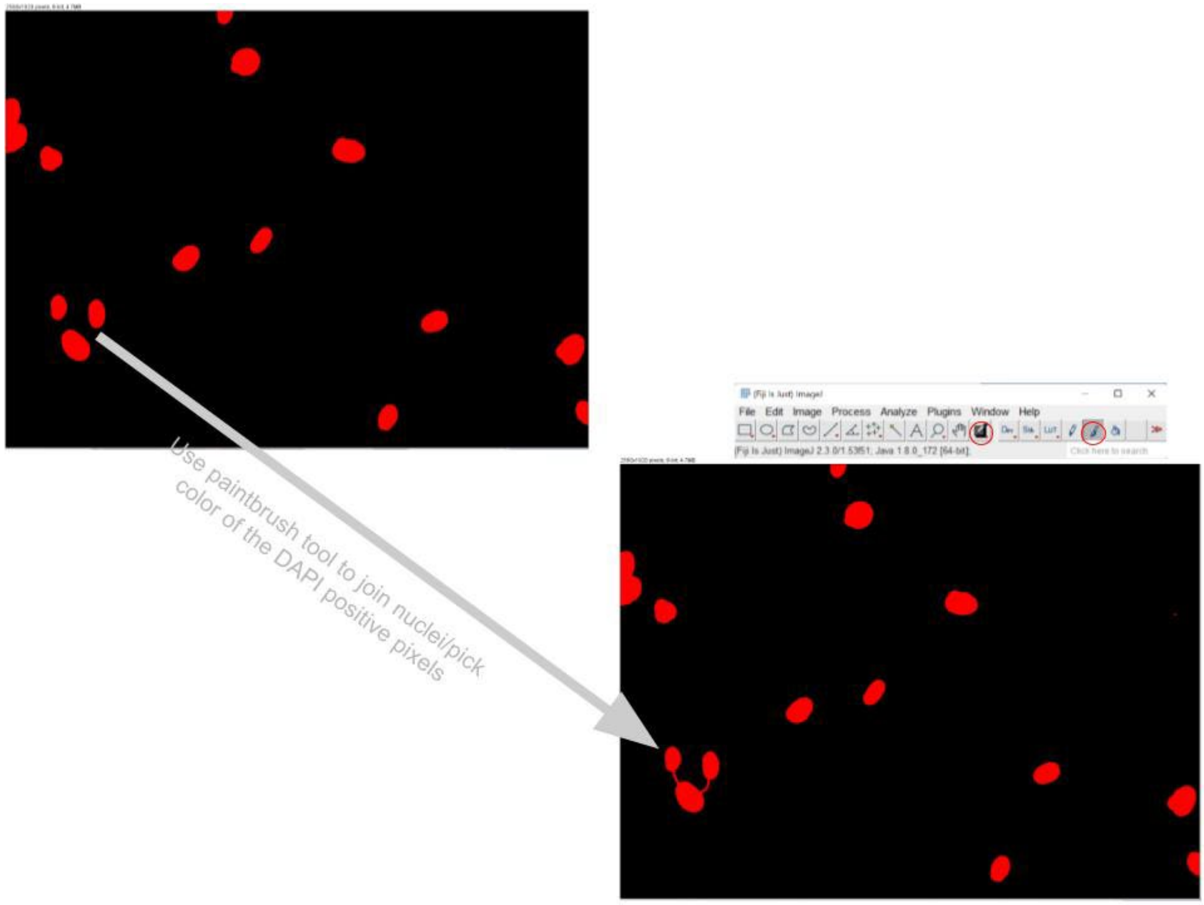

Press OK if all nuclei have been well selected

vi. Perform steps A-E on all folders containing images of DAPI staining

vii. After each folder is finished macro will automatically generate .xlsx file with results in the folders where selected images were.

4. Troubleshooting

Problem	Possible solution
**After setting the threshold I do not see difference between senescent cells and young cells**	
Early passage cells without senescence inductor display strong βGal positive signal	The staining conditions leading to saturation of the staining (wrong pH, too long incubation period with XGal)

Low level of βGal positive signal in senescent cells	Lack of senescence induction
I can visually see difference between senescent cells and early passage control but no difference in the results	The results are calculated as bGal signals per cell. In denser cultures bGal signal may seem to be more abundant than in early passage cells but when calculated per cell could turn out to be similar.
βGal positive signal from early passage cells are more than 10% of that of senescent cells	That value which we used in our research with VSMCs has been set experimentally and is true only for certain conditions and cell type. If your early passage control display βGal positive signal above that level it may still be OK as long as you are able to detect the difference. Else, consider changing the threshold or make sure you used the proper staining conditions.
**Macro doesn't run**	
Images do not load from the catalog	Image file format:<br><br>The macro extension will work only with .tif and .jpg files
Macro freeze or work slowly after processing few dozens of images	The analysis is taxing for system resources especially if significant numbers of images are being processed. It's especially true for older computers. In that case, split images to portions of arround 20 images and process them separately. Restart Fiji every 50 images.
After changing threshold values in the macro code the macro doesn't work anymore	Make sure you did not change other elements of the code besides the values themselves. Deleting even a single symbol will cause error.
Macro analyzed all images in the catalog but it displays error message at the end	Ignore it, it may occur if you repeat analysis of that same folder.

5. β-gal-analyser macro code

```
// beta gal analyzer macro developed by Adam Krzystyniak
// version 1.0
// part of the manuscript entitled: "Automatic tool for fast, and unbiased analysis of SA-
β-gal //activity in the cell culture that provides extra resolution compared to manual
analysis."

function color_t() {

	// Color Thresholder 2.3.0/1.53f
	// Autogenerated macro, single images only!

	min=newArray(3);
	max=newArray(3);
	filter=newArray(3);
	a=getTitle();
	run("HSB Stack");
	run("Convert Stack to Images");
	selectWindow("Hue");
	rename("0");
	selectWindow("Saturation");
	rename("1");
	selectWindow("Brightness");
	rename("2");
	min[0]=117;// change that value to min. "Hue" value from threshold window
after you set up the right threshold, default is 117
	max[0]=185;// change that value to max. "Hue" value from threshold window
after you set up the right threshold, default is 185
	filter[0]="pass";
	min[1]=80;// change that value to min. "Saturation" value from threshold window
after you set up the right threshold, default is 80
	max[1]=255;// change that value to max. "Saturation" value from threshold
window after you set up the right threshold, default is 255
	filter[1]="pass";
	min[2]=0;// change that value to min. "Brightness" value from threshold window
after you set up the right threshold, default is 0
```

```
	max[2]=255;// change that value to min. "Brightness" value from threshold
window after you set up the right threshold, default is 255
	filter[2]="pass";
	for (i=0;i<3;i++){
	  selectWindow(""+i);
	  setThreshold(min[i], max[i]);
	  run("Convert to Mask");
	  if (filter[i]=="stop")  run("Invert");
	}
	imageCalculator("AND create", "0","1");
	imageCalculator("AND create", "Result of 0","2");
	for (i=0;i<3;i++){
	  selectWindow(""+i);
	  close();
	}
	selectWindow("Result of 0");
	close();
	selectWindow("Result of Result of 0");
	rename(a);
	// Colour Thresholding-------------
}

// DAPI pos events count

function Count_positive_DAPI_cells_manual(directory) {

   run("8-bit");
	setAutoThreshold("Otsu dark");
	waitForUser("if threshold OK, press OK if not set threshold and press OK");
	run("Analyze Particles...", "size=200-Infinity circularity=0.10-1.00 add include");
	run("Set Measurements...", "area redirect=None decimal=5");
	numROIs = roiManager("count");
	roiManager("Measure");
	setResult("Number_of_cells",0, numROIs);
	path = directory+"no_of_cells.xlsx";
	run("Read and Write Excel", "file=["+path+"]");
	selectWindow("ROI Manager");
```

```
        run("Close");
        selectWindow("Results");
        run("Close");
        waitForUser("Close");
        run("Close");
}

// Quantify bGAL specific signal

function get_bGAL(directory) {

  run("Duplicate...", " ");

// on the duplicate make selection based on the color threshold

  color_t();

        run("Set Measurements...", "integrated redirect=None decimal=5");
        run("Measure");

// check if there is any bGAL signal above threshold on the image

        if_zero=getResult("IntDen", 0);

// if there is no signal above threshold create result entry equal to 0

        if(if_zero == 0){

                setResult("Area", 0, 0);
                setResult("Mean", 0, 0);
                setResult("StdDev", 0, 0);
                setResult("Perim", 0, 0);
                setResult("IntDen", 0, 0);
                setResult("Median", 0, 0);
                setResult("RawIntDen", 0, 0);

                path = directory+"bGAL.xlsx";
```

```
            //print(path);
            run("Read and Write Excel", "file=["+path+"]" );
            selectWindow("Results");
            run("Close");
            run("Close");
            waitForUser("No signal above threshold, press OK");
            run("Close");

        }

// if thre is bGAL signal on the image above the threshold analyse parameters of that signal

        else{
            selectWindow("Results");
            run("Close");
        run("Create Selection");
        roiManager("Add");
        roiManager("Save", directory + "RoiSet.zip");
        run("Close");
        //selectWindow("ROI Manager");
        run("Close");

        //      after closing the duplicate measure the pixel intensity

        run("8-bit");
        run("Invert");
        roiManager("Open", directory + "RoiSet.zip");
        run("Set Measurements...", "area mean standard perimeter integrated median redirect=None decimal=5");
            roiManager("Measure");
            path = directory+"bGAL.xlsx";
            //print(path);
            run("Read and Write Excel", "file=["+path+"]" );
            selectWindow("ROI Manager");
            run("Close");
            selectWindow("Results");
```

```
			run("Close");
			waitForUser("If OK press OK");
			run("Close");
		}
}

//chose directory with images of bGAL staining -> analyse bGAL specific signal in every
image

macro "bGAL_folder [a]" {

		dir=getDirectory("Choose a Directory");
		lst=getFileList(dir);
		File.delete(dir+"bGAL.xlsx");// delete any existing analysis in the folder not to
repeat already done analysis
		File.delete(dir+"RoiSet.zip");

		for ( i=0; i<lst.length; i++ ) {
		  if(endsWith(dir+lst[i], ".jpg"))
				open(dir+lst[i]);
		}

		for (i = 0; i < lst.length; i++) {
			get_bGAL(dir);
		}
}

//chose directory with images of DAPI staining -> count DAPI pos nuclei in every
image

macro "DAPI_folder [z]" {

		dir=getDirectory("Choose a Directory");
		lst=getFileList(dir);
		File.delete(dir+"no_of_cells.xlsx");// delete any existing analysis in the folder not
to repeat already done analysis
```

```
	for ( i=0; i<lst.length; i++ ) {
	  if(endsWith(dir+lst[i], ".tif")) //open .tif files
			open(dir+lst[i]);
	  if(endsWith(dir+lst[i], ".jpg"))//open .jpg files
			open(dir+lst[i]);
	}

	for (i = 0; i < lst.length; i++) {
		Count_positive_DAPI_cells_manual(dir);
	}
}

// module to facilitate setting up color threshold - interactive instructions

macro "set_color_threshold [q]" {
	waitForUser("Open file where you have positive bGAL signal and press OK");
	run("Color Threshold...");
	waitForUser("1) Create small selection arround positive signal\n2) Use sample button to sample positive signal\n3) Adjust Hue, Saturation and Brightness parametrs\nto obtain satisfactory selection of the bGAL signal\nDefault settings are:\nHue 117 - 175\nSaturation 80 - 255\nBrightness 0 - 255\nUse those values for setting threshold before you start analysis");
}
```
